# The Efficacy and Safety of CollaSel Pro^®^ Hydrolyzed Collagen Peptide Supplementation without Addons in Improving Skin Health in Adult Females: A Double Blind, Randomized, Placebo-Controlled Clinical Study Using Biophysical and Skin Imaging Techniques

**DOI:** 10.3390/jcm13185370

**Published:** 2024-09-11

**Authors:** Devrim Demir-Dora, Umut Ozsoy, Yilmaz Yildirim, Oguz Yilmaz, Peri Aytac, Beste Yilmaz, Emel Dogan Kurtoglu, Ayse Akman, Selim Tezman, Huseyin Serhat Inaloz, Aydin Erenmemisoglu

**Affiliations:** 1 Department of Medical Pharmacology, Faculty of Medicine, Akdeniz University, 07070 Antalya, Turkey; devrimdemirdora@akdeniz.edu.tr; 2 Department of Medical Biotechnology, Health Sciences Institute, Akdeniz University, 07070 Antalya, Turkey; 3 Department of Gene and Cell Therapy, Health Sciences Institute, Akdeniz University, 07070 Antalya, Turkey; 4 Department of Anatomy, Faculty of Medicine, Akdeniz University, 07070 Antalya, Turkey; ozsoyu@akdeniz.edu.tr (U.O.); yildirimyilmaz@akdeniz.edu.tr (Y.Y.); besteyilmaz29@gmail.com (B.Y.); 5 Department of Dermatology, Faculty of Medicine, Akdeniz University, 07070 Antalya, Turkey; oguzyilmazmd@gmail.com (O.Y.); aakman@akdeniz.edu.tr (A.A.); 6 Novagenix Bioanalytical Drug R&D Center, 06750 Ankara, Turkey; periaytac@gmail.com (P.A.); edogan@novagenix.com (E.D.K.); 7 Sel Sanayi Urunleri Ticaret ve Pazarlama AS, 34440 Istanbul, Turkey; stezman@tezmanholding.com; 8 Department of Dermatology, Faculty of Medicine, Gaziantep University, 27310 Gaziantep, Turkey; sinaloz@gantep.edu.tr; 9 Alpan Farma R&D Ltd., 38039 Kayseri, Turkey

**Keywords:** biophysical measurement, CollaSel Pro^®^, hydrolyzed collagen peptide, efficacy, safety, skin imaging

## Abstract

**Background**: This study aimed to evaluate the efficacy and safety of oral hydrolyzed collagen peptide (HCP) in healthy females by assessing the skin parameters via biophysical and skin imaging techniques. **Methods**: 112 females were randomly assigned to receive either HCP (*n* = 57; 10 g CollaSel Pro^®^) or placebo (*n* = 55; 10 g maltodextrin) daily for eight weeks. The contribution of HCP to skin elasticity, hydration, and roughness was investigated against a placebo, while the facial soft tissue sagging (RMS) and safety data were also recorded. **Results**: HCP was associated with significant improvements in skin elasticity (*p* = 0.009), skin hydration (*p* ranged from 0.003 to <0.001), and skin roughness (*p* ranged from 0.002 to <0.001). In the HCP vs. the placebo group, week eight values for skin elasticity (43.0 (7.4) vs. 40.3 (3.3) mPa, *p* = 0.017), skin hydration (65.8 (18.9) vs. 53.1 (14.9) g/m^3^, *p* < 0.001) and skin roughness (40.2 (20.4) vs. 24.9 (20.9) g/m^3^, *p* < 0.001) were significantly higher. In the HCP group, week 8 RMS values were significantly lower than baseline values (1.02 (0.21) vs. 1.10 (0.21) mm, *p* = 0.012). **Conclusions**: CollaSel Pro^®^ HCP can be considered a well-tolerated, safe product that effectively improves dermal health and the appearance of sagging and ameliorates the signs of the aging process.

## 1. Introduction

Collagen, the main structural protein in connective tissues, primarily contributes to the mechanical properties of tissues, such as the tensile strength in the skin and the resistance to traction in ligaments [1,2]. Specifically, type I and III collagen are crucial for skin health, maintaining the dermal layers’ strength, stability, hydration, and mechanical properties [1,3,4,5].

There are two types of skin aging: extrinsic, which is caused by stress, alcohol, smoking, sleep deprivation, and prolonged sun exposure, and intrinsic, which is caused by increasing age. Aging results in changes to the skin’s structure and appearance, including wrinkle development, the appearance of brown spots, and skin thickness [6]. The aging process has detrimental effects on connective tissue in the skin, including modifications in the structure and functionality of the extracellular matrix with a decrease in collagen fibers, elastin, and hyaluronic acid in the dermis, as manifested by decreased skin thickness and firmness, and loss of elasticity and hydration of the skin resulting in the appearance of fine lines, wrinkles and sagging [1,3,7,8,9].

Hydrolyzed collagen, obtained by denaturation of native collagen after an enzymatic breakdown of protein chains into small peptides, consists of segmented proteins with low molecular weight, enabling their distribution across several tissues quickly after digestion [9,10,11]. Accordingly, hydrolyzed collagen is enriched with amino acids (i.e., glycine, proline, and hydroxyproline), which help to form collagen fibers. In addition, it increases collagen deposition in the dermis and stimulates fibroblast proliferation and endogenous production of hyaluronic acid, collagen, and elastin [10,11,12,13]. Previous studies reported the favorable effects of hydrolyzed collagen on skin health in terms of improvements in skin wrinkles, elasticity, and hydration [2,12,14,15,16,17,18,19]. In addition, hydrolyzed collagen is a natural bioactive peptide with excellent biocompatibility, easy biodegradability, and weak antigenicity [12,20]. Therefore, it has become increasingly used in biomedical and cosmetic industries to improve mechanical properties (i.e., skin hydration and elasticity) and as a potential remedy for reverse skin aging by wrinkle reduction and skin rejuvenation [1,2,3,12,21,22]. In most of the previous studies, the use of liquid and solid forms of oral collagen supplements showed good patient tolerability and no adverse effects [15,23,24,25]. The United States Food and Drug Administration (FDA) has classified gelatin, from which collagen peptides are prepared, as a safe substance. Furthermore, based on the results of previous studies, both the World Health Organization (WHO) and the European Commission for Health and Consumer Protection have stated that hydrolyzed collagen is safe. Minor side effects, such as nausea, flatulence, or dyspepsia, can occur in some people ingesting collagen peptides [26,27].

Collagen is broadly available and isolated from several animal sources. In addition, numerous commercial collagen products are in food supplements or cosmetics [1,12]. However, while the popularity of oral collagen supplements (2000 Da) continues to rise, the exact effects of hydrolyzed collagen supplements (2000 Da) on the skin remain to be unclarified given the inconsistency between studies in collagen products (type, dose, added or not with vitamins or other ingredients with potential synergistic effect [minerals, antioxidants, coenzyme Q10, hyaluronic acid, and chondroitin sulfate]) used to produce the proposed effects on the skin [2,3,4,12].

This placebo-controlled randomized study aimed to evaluate the efficacy and safety of 12-week oral supplementation with a collagen product (CollaSel Pro^®^ hydrolyzed collagen peptide) in healthy females by assessing the skin parameters (elasticity, hydration, and roughness of the skin as well as facial configuration) via biophysical and skin imaging techniques.

## 2. Materials and Methods

### 2.1. Manufacturing and Quality Evaluation of Collagen

Collagen has been manufactured according to the Collagen Peptides Monograph for the European Union (EU) [28]. Bovine hide was used as a collagen source, and hydrolyzed collagen peptides Type I and Type III were extracted and purified from the bovine hide. The amino acid composition of hydrolyzed collagen peptide was determined as Alanine, Arginine, Aspartic acid, Glutamic acid, Glycine, Histidine, Hydroxyproline, Isoleucine, Leucine, Lysine, Ornithine, Phenylalanine, Proline, Serine, Threonine, Tyrosine, Valine and Methionine. The product was harmonious with all referred quality standards above mentioned monograph. All characterizations have been performed strictly following the necessary standards. Before submission to the Ethical Committee (EC) and Ministry of Health (MOH), all evaluations have been documented and detailed.

### 2.2. Study Design

One hundred and twenty female volunteers were included in this multiple-dose, randomized, double-blinded, placebo-controlled trial (ClinicalTrials.gov Identifier: NCT05235997) conducted between 31 January 2022 and 18 May 2022. The volunteers were randomly assigned by a statistician in 1:1 allocation to receive either collagen product (test product group; *n* = 57; a single oral dose of 10 g CollaSel Pro^®^ hydrolyzed collagen peptide) or placebo (placebo group; *n* = 55; oral maltodextrin) daily for eight weeks. The volunteers aged 35 to 60 years who had normal physical examination findings and normal blood pressure (SBP: 110–140 mmHg, DBP: 60–90 mmHg) and heart rate (50–100 bpm) values measured after 5 min of rest at the screening visit, not receiving any medication or supplementation which may interact with the study product or alter the study result, and those with ability to communicate adequately with the investigator and to comply with the study requirements, and gave consent to participate in the study were included in the study. Presence of atopic constitution or asthma and/or known allergy for collagen products and/or other any of the excipients of the product, hereditary problems of galactose intolerance, Lapp lactase deficiency or glucose–galactose malabsorption, cardiovascular, neurological, musculoskeletal, hematological, hepatic, gastrointestinal, renal, pulmonary, endocrinological, metabolism or psychiatric disease, any type of porphyria, history of drug abuse, difficulty of swallowing, malabsorption or any gastrointestinal surgery (except appendectomy or except herniotomy), the usage of oral retinoids or oral steroids in the 6 months prior to initiation of the study, use of topical retinoids, anti-wrinkle cosmetic products including retinol and/or alpha hydroxy acid (AHA), or moisture-rich cosmetic products or skincare therapy (via lasers or peeling) within the 3 months prior to initiation of the study and current intake of contraceptives, female hormones, obesity drugs, absorption inhibitors, antidepressants, or appetite suppressants or receiving a special diet due to any reason, or gain/loss of 5% of baseline weight and high probability of non-compliance to the study procedure and/or completion of the study according to the investigator’s judgment were the exclusion criteria of the study.

**Figure 1:**
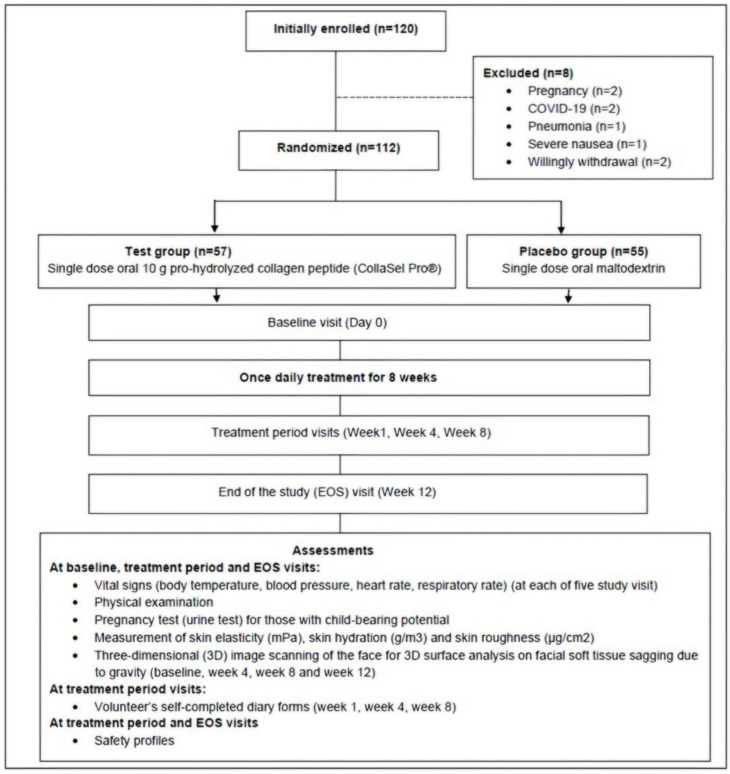
Study flowchart.

The final analysis was performed in 112 females with the exclusion of 8 females due to arousal of conditions consistent with exclusion criteria (pregnancy [*n* = 2], COVID-19 [*n* = 2], pneumonia [*n* = 1], severe nausea [*n* = 1]) and willingly withdrawal (*n* = 2) (Figure 1).

Written informed consent was obtained from each subject. The study was conducted following the ethical principles stated in the “Declaration of Helsinki” and approved by the Gaziantep University Clinical Research Ethics Committee (Date of Approval: 15 December 2021, Protocol No: 2021/03) and the Republic of Turkey Ministry of Health Turkish Medicines and Medical Devices Agency (Date of Approval: 4 January 2022, Protocol No: E-66175679-514.11.01-640037).

### 2.3. Data Collection

The study was based on five consecutive visits, including a baseline visit (day 0), the treatment period visits (week 1, week four, and week 8), and the end of the study (EOS) visit (week 12; 4 weeks after the 8-week-use of test product or placebo). Data on demographic characteristics (age, height, weight, and body mass index [BMI, kg/m^2^]) and medical history were recorded at baseline visits, along with explanations regarding filling out the volunteer’s diary forms and using the study product. In addition, data on vital signs (body temperature, blood pressure, heart rate, respiratory rate), physical examination, pregnancy test (urine test, for those with child-bearing potential), and measurement of skin elasticity (mPa: megapascal), skin hydration (g/m^3^) and skin roughness (μg/cm^2^) were recorded at each study visit (baseline and weeks 1, 4, 8 and 12) in the test product and placebo groups. The morphological changes in facial configuration (facial soft tissue sagging due to gravity between sitting and supine positions) were also evaluated by the three-dimensional (3D) scanning of the face and the surface analysis of images at baseline, week 8, and week 12 visits.

Data on the volunteer’s self-completed diary forms were recorded during the treatment period visits (weeks 1, 4, and 8). In contrast, the volunteer’s compliance to study drug administration, as well as the safety data on adverse events (AEs, reported by the volunteer or observed by the investigator), were recorded during the treatment period visits (weeks 1, 4 and 8) and at the EOS (week 12) visit. In addition, the contribution of hydrolyzed collagen peptide to the skin’s elasticity, hydration, roughness, and facial configuration was investigated against a placebo.

### 2.4. Test Product and Placebo

The test product was pro-hydrolyzed collagen peptide without add-ons (CollaSel Pro^®^, 10 g Sachet, Sel Sanayi Ürünleri Ticaret ve Pazarlama A.S, Istanbul, Turkey), while the placebo was 10 g maltodextrin, and each product was administered once daily for eight weeks.

### 2.5. Measurement of Skin Elasticity, Skin Hydration, and Skin Roughness

Skin elasticity, hydration, and roughness measurements were performed at five time points (baseline, week 1, week 4, week eight, and week 12) with a Callegari Soft-Plus (Callegari Srl, Parma, Italy) device designed for the assessment of skin conditions.

The skin elasticity (megapascal, mPa) was measured from the crow’s feet area and inner forearm using the elasticity probe of the device and based on a stress/deformation method by skin suction with higher values indicating improved skin elasticity.

The skin hydration (g/m^3^) was measured from the crow’s feet area and inner forearm using the specific probe of the device, which is pressed down sufficiently to keep the pressure constant until the measurement while the whole sensor is in contact with the skin and is based on the hydration of the stratum corneum and the amount of electric current passing through a capacitor. Higher values were considered to indicate improved skin hydration.

Skin roughness (μg/cm^2^) was measured from the crow’s feet area based on the image captured using a video camera probe of the device, which determines the depth and width of the wrinkles in the crow’s feet area. Higher values were considered to indicate amelioration of skin roughness.

### 2.6. 3D Facial Scanning and Image Processing

Facial scanning was performed with an Artec EVA 3D Light Scanner (Artec Group 2013, Luxembourg). The scanning (speed: 15 frames per second; depth of the scanning field: 400 mm for near and 1000 mm for far; work distance: 0.4–1 m; three-dimensional (3D) accuracy: up to 0.1 mm; 3D resolution: up to 0.5 mm) was performed in the sitting (seated on a chair) and supine (lying on a stretcher) positions with the volunteers’ heads positioned for the Frankfort horizontal plane to be parallel to the ground or perpendicular to the stretcher, respectively. To avoid motion artifacts, the volunteers were asked to keep neutral facial expressions, mouth closed, and teeth in total occlusion and remain still during scanning. 

The 3D surfaces were created by Artec Studio 11 software (version 11.2.2.16; Artec Group, Luxembourg, licensed) in STL file format. The scanned masks of each subject were imported into the same workspace of Artec Studio 11 Software. Each scan was oriented manually according to the established coordinate system (*x*-axis, left/right; *y*-axis, superior/inferior; Z-axis, anterior/posterior) using translation and rotation in all three directions. The common origin of the three axes was set at a mid-endocanthion point (a point halfway between the inner corners of the eyes), as previously reported to show minimal positional change over time [29]. Unwanted extraneous data, such as the ears and the hairline, were excluded. Finally, the software automatically aligned the facial masks.

### 2.7. Surface Analysis for Facial Soft Tissue Sagging

After obtaining digital face masks from sitting and supine positions superimposed by Artec Studio 11 Software, the distance between the sitting and supine masks was computed by the same software, and colored distance maps were created for quantitative surface analyses. Morphological changes in facial soft tissue due to the effect of gravity after the change in head position were assessed by calculating the average of the displacements between sitting and supine positions at each vertex in millimeters using the root mean square (RMS) formula. The increase in RMS value between sitting and supine positions indicates the increase in the sagging of facial soft tissue caused by gravity and vice versa. For more detail, the related study can be reviewed [30].

### 2.8. Statistical Analysis

At least 106 subjects (53 for each group) were calculated to be included in this superiority study via sample size estimation based on a power of 80% at a type I error of 0.05, assuming the expected percentage of improvement (percent change from baseline) of 20% in the test product group and 5% in the placebo group. Considering the potential dropouts, 120 subjects (60 subjects for each group) were initially enrolled in the study, whereas due to the exclusion of 8 subjects, 112 subjects were included in the final analysis.

Statistical analysis was performed using R Project. The independent-sample *t*-test or a Mann–Whitney U test was used to analyze parametric variables, while the paired *t*-test or Wilcoxon test was used to analyze change over time within a group. Three-dimensional facial scanning images were analyzed with GraphPad Prism version 9.0.0 software (GraphPad, La Jolla, CA, USA). Data were expressed as “mean (standard deviation, SD)”, median (minimum–maximum), and percentage (%) where appropriate. *p* < 0.05 was considered statistically significant.

## 3. Results

### 3.1. Baseline Characteristics (n = 112)

Overall, the mean age of female subjects was 44.4 years (SD 5.9, range 35.0 to 57.0 years). The mean BMI was 25.5 kg/m^2^ (SD 3.8, range 18.8 to 36.2 kg/m^2^). Overall, 62 (55.4%), 34 (30.4%), and 16 (14.2%) females were in the average weight (BMI 18.5–24.9 kg/m^2^), overweight (BMI 25–29.9 kg/m^2^) and obese (BMI > 30 kg/m^2^) categories based on BMI values.

### 3.2. Skin Elasticity in Test Product and Placebo Groups

**Figure 2:**
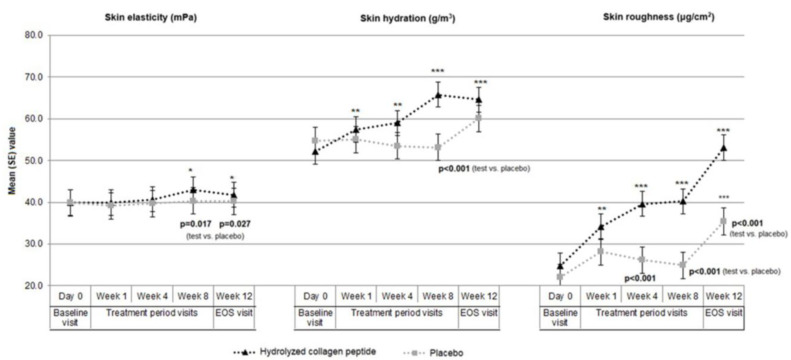
Skin elasticity (mPa), skin hydration (g/m^3^), and skin roughness (μg/cm^2^) values in test product and placebo groups during the study period. * *p* < 0.05, ** *p* < 0.01 and *** *p* < 0.001 compared to baseline measurement in each group.

In the test product (CollaSel Pro^®^ hydrolyzed collagen peptide) group, skin elasticity was significantly improved at week 8 (43.0 (7.4) mPa, *p* = 0.009 and *p* = 0.006, respectively) and week 12 (41.8 (4.3) mPa, *p* = 0.001 and *p* = 0.01, respectively) visits compared to baseline (39.9 (4.7) mPa) and week 1 (39.9 (5.2) mPa) values. The week eight values were also significantly higher than week 4 (40.7 (6.0) mPa, *p* = 0.049). No significant change was noted in skin elasticity during the study period in the placebo group (Table 1, Figure 2).

The skin elasticity values at week 8 (43.0 (7.4) vs. 40.3 (3.3) mPa, *p* = 0.017) and week 12 (41.8 (4.3) vs. 40.2 (3.3) mPa, *p* = 0.027) were significantly higher in the test product (CollaSel Pro^®^ hydrolyzed collagen peptide) group than in the placebo group (Table 1, Figure 2).

### 3.3. Skin Hydration in Test Product and Placebo Groups

In the test product (CollaSel Pro^®^ hydrolyzed collagen peptide) group, skin hydration was significantly improved starting from week 1, with significantly higher values at week 1 (57.4 (17.8) g/m^3^, *p* = 0.003), week 4 (59.0 (18.2) g/m^3^, *p* = 0.002) week 8 (65.8 (18.9) g/m^3^, *p* < 0.001) and week 12 (64.6 (14.4) g/m^3^, *p* < 0.001) compared to baseline values (52.2 (16.1) g/m^3^). Skin hydration values were also significantly higher at week eight and week 12 visits compared to week 1 (*p* < 0.001 for each) and week 4 (*p* < 0.001 and *p* = 0.004, respectively) (Table 1, Figure 2).

In the placebo group, no significant change was noted in skin hydration values during the 8-week treatment period, whereas EOS (week 12) values (60.1 (15.8) g/m^3^) were significantly higher than baseline (54.8 (14.2) g/m^3^, *p* = 0.017), week 1 (55.0 (12.5) g/m^3^, *p* = 0.03), week 4 (53.5 (14.1) g/m^3^, *p* = 0.005) and week 8 (53.1 (14.9) g/m^3^, *p* = 0.002) values (Table 1, Figure 2). 

The skin hydration values at week 8 were significantly higher in the test product (CollaSel Pro^®^ hydrolyzed collagen peptide) group than in the placebo group (*p* < 0.001) (Table 1, Figure 2).

### 3.4. Skin Roughness in Test Product and Placebo Groups

In the test product (CollaSel Pro^®^ hydrolyzed collagen peptide) group, skin roughness was significantly ameliorated starting from week 1, with significantly higher values at week 1 (mean (SD) 34.1 (21.8) μg/cm^2^, *p* = 0.002), week 4 (39.6 (15.8) μg/cm^2^, *p* < 0.001), week.

8 (40.2 (20.4) μg/cm^2^, *p* < 0.001) and week 12 (53.1 (24.1) μg/cm^2^, *p* < 0.001) when compared to baseline (24.8 (18.2) μg/cm^2^) values. In addition, the skin roughness values were also significantly higher at week 8 (*p* = 0.033) and week 12 (*p* < 0.001) visits compared to week one values, and at week 12 visit compared to week 4 (*p* < 0.001) and week 8 (*p* < 0.001) values (Table 1, Figure 2).

In the placebo group, skin roughness values were also higher at week 1 (28.1 (16.3) μg/cm^2^, *p* < 0.001), week 4 (26.1 (16.3) μg/cm^2^, *p* = 0.044), and week 12 (35.4 (25.6) μg/cm^2^, *p* < 0.001) compared to baseline (22.0 (13.8) μg/cm^2^) values. In addition, the skin roughness values in the placebo group were also higher at week four compared to week 1 (*p* = 0.024) and at week 12 compared to week 4 (*p* = 0.002) and week 8 (*p* < 0.001) values (Table 1, Figure 2).

The skin roughness values at week 4, week eight, and week 12 were significantly higher in the test product (CollaSel Pro^®^ hydrolyzed collagen peptide) group than in the placebo group (*p* < 0.001 for each) (Table 1, Figure 2).

### 3.5. 3D Surface Analysis for Facial Configuration in Test Product and Placebo Groups

**Figure 3:**
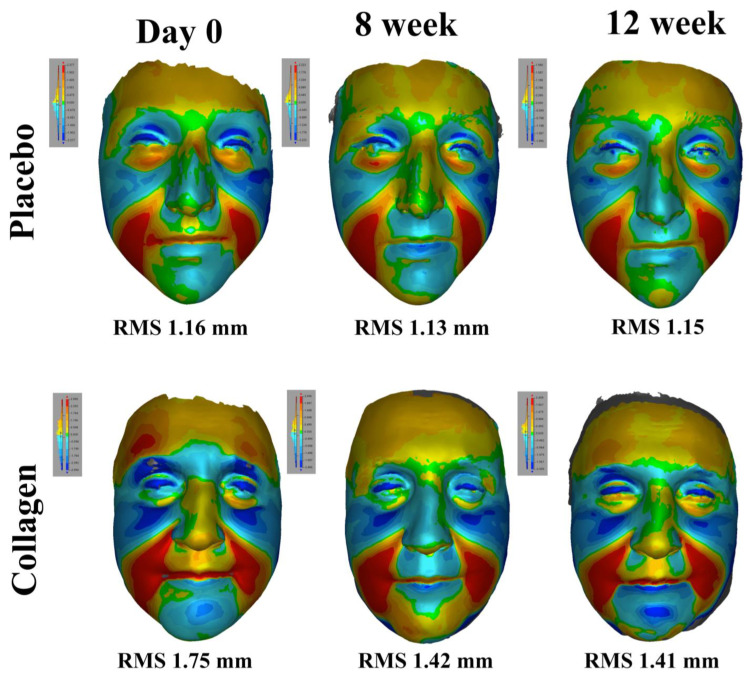
The Figure shows gravity-induced sagging on the facial soft tissue, with a colored deviation map in the placebo and collagen groups. Furthermore, sagging in the volunteers’ faces for the relevant week is stated quantitatively with the RMS values at the bottom of the Figure. Finally, the color scale of each measurement is presented in the upper corner of the Figure. The green color (zero) on the scale indicates no sagging and red and dark blue indicate sagging in different axes. Accordingly, note the red and dark blue colors on the forehead and around the eyes on day 0 in the collagen group. In the eighth week, the colors were turned into colors closer to zero on the scale. At the end of 12 weeks, the change was preserved. A similar change was not observed in the placebo group.

Three-dimensional surface analysis was available for 28 females in the test product group and 25 in the placebo group. The significant decrease in RMS values (indicating a decrease in sagging of facial soft tissue caused by gravity) was noted at week eight compared to baseline (1.02 (0.21) vs. 1.10 (0.21) mm, *p* = 0.012) in the test product (CollaSel Pro^®^ hydrolyzed collagen peptide) group. The improvement from baseline values was also evident at week 12 when the extreme values were excluded (1.03 (0.22) vs. 1.12 (0.22) mm, *p* = 0.009). No significant change was noted in RMS values in the placebo group during the study period. Also, no significant change was noted between the test product and placebo groups regarding RMS values (Table 2, Figure 3).

### 3.6. Safety Data

A total of 43 AEs (most commonly nausea [20.9%] and constipation [9.3%]) were reported in 19 subjects. Possible relation to test product was reported for 32 (74.4%) of 43 AEs, 95.3% of AEs were mild AEs, and no serious adverse events (SAEs) were reported (Table 3).

## 4. Discussion

This randomized double-blinded placebo-controlled trial in female volunteers revealed the efficacy and safety of 8-week once-daily continuous use of hydrolyzed collagen peptide (Collasel Pro^®^) in terms of significantly improved skin parameters (elasticity, hydration, and roughness). The test product (CollaSel Pro^®^ hydrolyzed collagen peptide) was found to be superior to the placebo in improving skin elasticity (in the 8th and 12th weeks), skin hydration (in the eighth week), and skin roughness (at the 4th and 8th, and 12th weeks). In addition, our results with the 3D facial soft tissue analysis method revealed that regular use of hydrolyzed collagen peptide significantly reduced gravity-induced sagging of facial soft tissue.

Oral ingestion of hydrolyzed collagen, as a nutraceutical supplement, has been consistently reported to improve mechanical properties of the skin, such as elasticity, skin hydration, and trans-epidermal water loss and to increase dermal density and reducing facial wrinkles, by increasing the circulatory levels of collagen-derived peptides and enhancing the production of fibroblast, elastin and glycosaminoglycans [2,12,14,15,16,17,18,19]. In most of these placebo-controlled studies, the significant improvement in skin parameters (elasticity, firmness, texture, and hydration or wrinkles) and the recovery of the degenerative changes of the extracellular matrix have been reported based on 12-week use of hydrolyzed collagen in combination with skin enhancing nutrients such as 3 g hydrolyzed collagen peptide (plus vitamin C or astaxanthin), 10 g collagen peptide (plus vitamins A, C, and E and zinc), 50 mL hydrolyzed collagen (plus hyaluronic acid and N-acetylglucosamine, borage oil, vitamins, minerals antioxidants, and additional bioactive ingredients) or 30-mL of fish-derived collagen (plus ornithine) [4,10,12,14,31,32,33,34]. However, despite the likelihood of the synergistic contribution of these add-ons in the overall beneficial effects achieved, the positive outcomes of supplementation were solely attributed to collagen without comparatively assessing the effect of add-ons [2,12,15].

In this regard, the association of CollaSel Pro^®^, as a pure hydrolyzed bovine collagen peptide formulation, with improved skin elasticity, hydration, and roughness, as well as the facial contours in our study, seems to strongly suggest the positive outcomes of the hydrolyzed collagen product per se, excluding any potential synergistic effects of skin enhancing nutrients on the improvement of skin mechanical properties. Furthermore, the favorable effects of the CollaSel Pro^®^ appear even after 1-week use for skin hydration and roughness and after at least 8-week use for skin elasticity and facial configuration.

Similarly, placebo-controlled studies with 1 g or 5 g hydrolyzed collagen revealed significant improvement in skin hydration as early as after six weeks of intake and improved skin elasticity, skin texture, and wrinkles after 12 weeks of intake [10,12,35], while those with 10 g hydrolyzed collagen supplementation indicated improvements in skin hydration and facial wrinkles after four weeks or 12 weeks of intake [4,14,36,37,38]. Studies with the use of different doses of fish-hydrolyzed collagen (2.5 g, 5 g, or 10 g) and hydrolyzed porcine collagen (2.5 or 5 g) vs. placebo in healthy women indicated improvement in the hydration of the skin only in the 5 g and 10 g treated groups for fish hydrolyzed collagen [39] and improvement in the skin elasticity after eight weeks of intake in both 2.5 g and 5 g groups for hydrolyzed porcine collagen [19].

Notably, other commercial collagen products such as Pure Gold Collagen^®^ (type I hydrolyzed collagen product extracted from fish), BioCell Collagen^®^ (type II hydrolyzed collagen), VERISOL^®^ (type I porcine hydrolyzed collagen), and Vinh Wellness Collagen^®^ (hydrolyzed marine collagen) also revealed significantly improved skin properties after regular use [1,18,19,40,41,42].

Once daily (50 mL) use of Pure Gold Collagen^®^ Food Supplement (Minerva Research Labs Ltd., London, UK) was reported to be associated with a reduction in the dryness of the skin, the wrinkles, and the nasolabial depth after 60 days of intake, while the increase in skin firmness and collagen density was noted after 12 weeks of intake [18]. The 12-week use of a 1 g daily or twice daily BioCell Collagen^®^ (BCC; BioCell Technology, LLC, Irvine, CA, USA) was reported to reveal a significant reduction in skin dryness and wrinkles along with improved tone, texture, and hyperpigmentation of the skin. In contrast, dermal hemoglobin and collagen content was also reported to increase after six weeks of intake, emphasizing the likelihood of physiological changes in the dermal and epidermal layers besides the visible changes in the skin [41]. The daily use of 2.5 or 5 g bioactive collagen peptide VERISOL^®^ (Gelita AG, Eberbach, Germany) for four weeks in healthy women was reported to improve the skin elasticity and reduce the eye wrinkle formation along with the increase in the biosynthesis of procollagen I, elastin, and fibrillin, which was maintained after the discontinuation of the treatment [19,42].

The later improvement of skin elasticity and facial configuration than the skin hydration and roughness in our female subjects (mean age 44.4 years, range, 35.0 to 57.0 years) seems notable given the likelihood of improvement in skin elasticity to take longer depending on the age of subjects and aging effect on loss in elasticity [17,43]. The 12-week once-daily use of a Vinh Wellness Collagen (VWC) vs. placebo in women (aged 45–60 years) was reported to be associated with more significant improvements in overall skin elasticity, hydration, radiance, firmness, and wrinkle score with no safety or intolerance issues. In conclusion, they suggested that fish-derived hydrolyzed collagen can be safely used to improve skin health in an aging population [14]. In a study with female subjects diagnosed with dry and rough skin, six weeks of daily intake of 7 g of fish hydrolyzed collagen was reported to significantly improve the moisture content of the skin in face-cheek, forearm, and the back of the neck and enhance the skin elasticity by reducing wrinkles and lowering skin roughness [44]. In another place-controlled study, the use of 2.5 or 5 g hydrolyzed collagen for 60 days was reported to improve skin elasticity significantly and skin moisture compared to placebo, while the improvement in elasticity was noticed among older women relatively earlier (at 1-month follow-up) [19]. The collagen content and treatment duration have also improved elasticity and wrinkles [12]. The oral ingestion of 5 g hydrolyzed collagen derived from fish source (higher and lower peptide content) in females for eight weeks was reported to significantly improve the facial skin elasticity and skin hydration, while the high-content collagen yielded the best results in skin elasticity as well as for surface skin measurements with a significant reduction in the number of wrinkles, wrinkle area, wrinkle depth, and roughness compared to the placebo group [45]. The oral ingestion of 2.5 g porcine type I collagen peptides for eight weeks was reported to improve the eye wrinkle volume parameter after four weeks of treatment; after eight weeks of intake, the improvement in skin health was reported to be more pronounced with the reduction of wrinkles up to 20%, increase in production of procollagen type I and elastin by up to 65% and 18%, respectively, and increase in fibril content by up to 6% compared to the placebo [42].

The 3D surface analysis in our study revealed the improved facial configuration with a decrease in facial soft tissue sagging due to gravity after the 8-week use of CollaSel Pro^®^ in healthy females. Likewise, daily oral supplementation with hydrolyzed fish collagen (plus vitamins and antioxidants) vs. placebo for 90 days was reported to improve the morphological and structural characteristics of the dermis with positive changes in the skin architecture and improvement in collagen fiber organization on histological analysis of skin biopsies and improvement in skin texture, firmness, and appearance on the high-resolution image analysis [2,17,46]. The use of hydrolyzed collagen (plus antioxidants; Nutrova^®^) for 60 days in healthy women was reported to improve metrics in improving skin health based on 3D image reconstruction for analysis of skin topography and to reduce wrinkle width, open pores, skin roughness, in addition to improved skin hydration, firmness, and barrier function [47]. The use of hydrolyzed collagen (plus vitamins) vs. placebo once daily for 12 weeks in females (40–60 years) was reported to be associated with improved dermis echogenicity and elasticity and viscoelasticity of the skin on the high-frequency ultrasound imaging, as well as a reduction of wrinkles and the total amount of pores on the skin [4]. The increase in the dermis echogenicity after 90 days of treatment indicates the efficacy of hydrolyzed collagen in increasing fibroblast density, enhancing the formation of collagen fibrils and thus slowing the aging process by improving the density, firmness, and elasticity of the skin [4,48]. In a study among 294 volunteers (aged 18–74 years) by 40 dermatologists across five different countries, once daily use of 50 mL hydrolyzed collagen from tilapia and pangasius fish (plus hyaluronic acid, vitamins, and minerals) was reported to be associated with visible or significant improvement in the facial lines, facial photoaging problems, and skin dryness after 60 days of treatment, with an increment in dermal collagen density after 12 weeks of treatment and increased skin firmness after 130 days of treatment [49]. In a placebo-controlled study of female volunteers (40–59 years old) recruited to ingest 10 g of either fish collagen, porcine collagen, or placebo, porcine collagen showed the best hydration, increasing by up to 28% after eight weeks of intake. In comparison, the high-frequency ultrasound findings revealed that oral intake of collagen peptides increased dermal echogenicity significantly immediately after four weeks of treatment. The effect was maintained after 12 weeks of treatment [16].

The current study’s favorable safety and tolerability profile of 10 g daily CollaSel Pro^®^ hydrolyzed collagen peptide agrees with the consistently reported safety of dietary hydrolyzed collagen supplements with no severe or profound adverse effects [3,10,12,19,35,45,50]. Several systemic reviews and meta-analyses reported hydrolyzed collagen supplementation’s efficacy and safety in improving females’ skin health [2,3,15]. In a systematic review and meta-analysis of 19 randomized controlled trials (RCTs) in 1125 participants (aged 20–70 years, 96% women) regarding the effects of hydrolyzed collagen supplementation on skin health, the authors concluded favorable anti-aging effects of 90-day hydrolyzed collagen supplementation compared with placebo in terms of improved skin hydration and elasticity, and reduced wrinkles [15]. In another systematic review of 5 RCTs assessing the impact of collagen supplementation on skin appearance, hydrolyzed collagen supplements were concluded to improve the signs of skin aging by decreasing facial wrinkles and improving skin hydration and elasticity in addition to the texture, firmness, and appearance of the skin and the supplement intake is considered effective and safe with no significant adverse effects [2]. In a systematic review of the 12 studies, oral collagen supplements were considered to improve skin elasticity, turgor, and hydration and reduce skin wrinkling and roughness without side effects [3].

In most of the previous studies, oral collagen supplements showed very well tolerability and no adverse effects [15,23]. In another clinical study for tolerability and efficacy assessment on collagen supplements, a few gastrointestinal (GI) complications were observed [24]. In a clinical study conducted by Guadanhim et al. on postmenopausal women aged 60–93, the primary outcomes included an increase in dermal collagen I and elastin content, and epidermal thickness after 24 weeks. Additionally, the study reported an increase in dermal echogenicity, thickness, and pixel intensity of the total and upper dermis in ultrasound images, improvements in skin viscoelastic measures, and a reduction in Dermatology Life Quality Index (DLQI) scores after 12 and 24 weeks. No adverse effects were observed in the study [25]. Our data were harmonious with these studies.

Skin aging is associated with reduced production of fibroblasts and increased expression of matrix metalloproteinases, which leads to increased degradation of collagen and elastin fibers (as the primary network supporting the skin’s structure and smooth appearance) and the consequent physiological changes at the dermis level are manifested by visible signs such as dryness, laxity, and wrinkles in the face [12,17,51]. Daily intake of collagen peptides decreases the expression levels of matrix metalloproteinase, responsible for collagen breakdown, and enhances fibroblasts’ growth and proliferation [12,52,53,54]. In a systematic review of 19 publications focusing on mechanisms of action and the effects of collagen supplements on skin health parameters in healthy subjects, oral administration of intact or hydrolyzed collagen was concluded to improve clinical manifestation of skin health based on three different mechanisms including the direct effects of collagen peptides on fibroblasts, the M2-like macrophages, and the oral tolerance-related mechanisms [55]. Nonetheless, while products of hydrolyzed collagen peptides are considered anti-aging remedies, most studies are based on 12–24 weeks of follow-up, and types and doses of collagen were not similar across the studies [3]. Accordingly, longer-term studies with consistent types and doses of hydrolyzed collagen in different settings are needed to explore further the potential adverse effects in the long run [3].

The major strength of our study seems to be the demonstration of skin health effects of oral collagen supplementation using a pure hydrolyzed collagen peptide not combined with other potentially adequate nutrients and based on a comprehensive analysis of biophysical and 3D imaging techniques. However, certain limitations to this study should be considered. First, the relatively small sample size and lack of data on 3D surface analysis in at least half of the subjects is an important limitation to the more accurate assessment of the changes in skin parameters in test product vs. placebo groups. Second, a lack of data on histological analysis of skin biopsies and a relatively shorter follow-up are other limitations that otherwise would extend the knowledge achieved in the current study.

## 5. Conclusions

In conclusion, our findings indicate that CollaSel Pro^®^ hydrolyzed collagen peptide is an effective collagen product without add-ons in improving skin elasticity, hydration, roughness, and facial configuration in females after continuous use. Furthermore, the favorable effects of the product appear even after 1-week use for skin hydration and roughness and after at least 8-week use for skin elasticity and facial configuration. Accordingly, CollaSel Pro^®^ hydrolyzed collagen peptide can be considered a well-tolerated, safe product that effectively improves dermal health and ameliorates the signs of skin aging. However, further research over extended durations is needed to evaluate the long-term use of hydrolyzed collagen peptides to comprehensively understand the potential effects of collagen on the aging process.

## Figures and Tables

**Table 1:** Skin measurements during study visits in test product (CollaSel Pro^®^ hydrolyzed collagen peptide) and placebo groups.

		Test Product (Hydrolyzed Collagen Peptide) Group (*n* = 57)					Placebo Group (*n* = 55)				
		Baseline Visit	Treatment Period Visits			EOS Visit	Baseline Visit	Treatment Period Visits			**EOS Visit**
Skin Measurements		Day 0	Week 1	Week 4	Week 8	Week 12	Day 0	Week 1	Week 4	**Week 8**	**Week 12**
**Skin elasticity (mPa)**	*n*	57	57	57	57	56	55	54	55	55	55
	mean(SD)	39.9 (4.7)	39.9 (5.2)	40.7 (6.0)	43.0 (7.4)	41.8 (4.3)	39.9 (6.3)	39.1 (6.0)	39.7 (5.0)	40.3 (3.3)	40.2 (3.3)
*p*-value	vs. baseline		0.883	0.222	**0.009**	**0.001**		0.251	0.753	0.571	0.693
	vs. week 1			0.237	**0.006**	**0.01**			0.218	0.078	0.066
	vs. week 4				**0.049**	0.14				0.298	0.392
	vs. week 8					0.175					0.754
*p*-value vs. placebo		0.971	0.494	0.328	**0.017**	**0.027**					
**Skin hydration (g/m^3^)**	*n*	57	57	57	57	56	55	54	55	55	55
	mean(SD)	52.2 (16.1)	57.4 (17.8)	59.0 (18.2)	65.8 (18.9)	64.6 (14.4)	54.8 (14.2)	55.0 (12.5)	53.5 (14.1)	53.1 (14.9)	60.1 (15.8)
*p*-value	vs. baseline		**0.003**	**0.002**	**<0.001**	**<0.001**		0.846	0.47	0.467	**0.017**
	vs. week 1			0.47	**<0.001**	**<0.001**			0.329	0.285	**0.03**
	vs. week 4				**0.001**	**0.004**				0.835	**0.005**
	vs. week 8					0.336					**0.002**
*p*-value vs. placebo		0.367	0.412	0.076	**<0.001**	0.115					
**Skin roughness (μg/cm^2^)**	*n*	53	56	56	56	56	53	51	55	55	55
	mean(SD)	24.8 (18.2)	34.1 (21.8)	39.6 (15.8)	40.2 (20.4)	53.1 (24.1)	22.0 (13.8)	28.1 (16.3)	26.1 (16.3)	24.9 (20.9)	35.4 (25.6)
*p*-value	vs. baseline		**0.002**	**<0.001**	**<0.001**	**<0.001**		**<0.001**	**0.044**	0.235	**<0.001**
	vs. week 1			0.08	**0.033**	**<0.001**			**0.024**	0.115	0.076
	vs. week 4				0.447	**<0.001**				0.687	**0.002**
	vs. week 8					**0.001**					**<0.001**
*p*-value vs. placebo		0.371	0.111	**<0.001**	**<0.001**	**<0.001**					

EOS: End of study, *p*-values < 0.05 are written in bold.

**Table 2:** Three-dimensional surface analysis on facial soft tissue sagging.

		Test Product Group			Placebo Group		
		Baseline Visit	Treatment Visit	EOS Visit	Baseline Visit	Treatment Visit	EOS Visit
3D Surface Analysis		Day 0	Week 8	Week 12	Day 0	Week 8	Week 12
**RMS (mm)**	*n*	28	28	28	25	23	25
	mean(SD)	1.10 (0.21)	1.02 (0.21)	1.09 (0.20)	1.08 (0.25)	1.03 (0.15)	1.11 (0.24)
*p*-value	vs. baseline		**0.012**	0.546		0.659	0.493
	vs. week 8			**0.017**			0.605
*p*-value vs. placebo		0.493	0.532	0.894			
RMS (mm) *	*n*	23	23	23	20	18	20
	mean(SD)	1.12 (0.22)	1.03 (0.22)	1.06 (0.21)	1.1 (0.27)	1.03 (0.15)	1.06 (0.24)
*p*-value	vs. baseline		**0.009**	**0.027**		0.94	0.48
	vs. week 8			0.23			0.47

RMS: Root mean square (RMS); EOS: End of study; * after the exclusion of subjects with extremely high RMS values in the test product (*n* = 5) and placebo (*n* = 5) groups. *p*-values < 0.05 are written in bold.

**Table 3:** Safety data.

**Patients with adverse events (N)**	19
**Number of SAEs (*n*)**	0
**Number of AEs (*n*)**	43
**Type of AEs, *n*(%)**	
Nausea	9 (20.9)
Constipation	4 (9.3)
Mild itching	2 (4.7)
Menstrual bleeding increase	2 (4.7)
Oedema	2 (4.7)
Dry mouth	2 (4.7)
Bloating	2 (4.7)
Skin tension increased	2 (4.7)
Weight increase	2 (4.7)
Vomiting	2 (4.7)
Halitosis	1 (2.3)
Dry skin	1 (2.3)
Fatigue	1 (2.3)
Heat	1 (2.3)
Diarrhea	1 (2.3)
Abdominal pain	1 (2.3)
Thirst	1 (2.3)
Mild tingling	1 (2.3)
Headache	1 (2.3)
Rash	1 (2.3)
Drowsiness	1 (2.3)
Acne	1 (2.3)
Cracked nail	1 (2.3)
State of repletion	1 (2.3)
Total	43 (100.0)
**Relation to study drug, *n*(%)**	
Certain	-
Probable/Likely	-
Possible	32 (74.4)
Unlikely	11 (25.6)
**Severity**	
Mild	41 (95.3)
Moderate	2 (4.7)
Severe	0 (0.0)

AE: Adverse event; SAE: Serious adverse events.

## Data Availability

The datasets generated and/or analyzed during the current study are available from the corresponding author upon reasonable request.
